# Effect of Electrode Distance and Size on Electrocorticographic Recordings in Human Sensorimotor Cortex

**DOI:** 10.1007/s12021-024-09689-z

**Published:** 2024-10-09

**Authors:** Simon H. Geukes, Mariana P. Branco, Erik J. Aarnoutse, Annike Bekius, Julia Berezutskaya, Nick F. Ramsey

**Affiliations:** Department of Neurology and Neurosurgery, University Medical Center Utrecht Brain Center, Utrecht University, P.O. Box 85500, 3508 GA Utrecht, The Netherlands

**Keywords:** Electrocorticography, Sensorimotor cortex, Ultra-high-density ECoG, Intraoperative ECoG

## Abstract

Subdural electrocorticography (ECoG) is a valuable technique for neuroscientific research and for emerging neurotechnological clinical applications. As ECoG grids accommodate increasing numbers of electrodes and higher densities with new manufacturing methods, the question arises at what point the benefit of higher density ECoG is outweighed by spatial oversampling. To clarify the optimal spacing between ECoG electrodes, in the current study we evaluate how ECoG grid density relates to the amount of non-shared neurophysiological information between electrode pairs, focusing on the sensorimotor cortex. We simultaneously recorded high-density (HD, 3 mm pitch) and ultra-high-density (UHD, 0.9 mm pitch) ECoG, obtained intraoperatively from six participants. We developed a new metric, the normalized differential root mean square (ndRMS), to quantify the information that is not shared between electrode pairs. The ndRMS increases with inter-electrode center-to-center distance up to 15 mm, after which it plateaus. We observed differences in ndRMS between frequency bands, which we interpret in terms of oscillations in frequencies below 32 Hz with phase differences between pairs, versus (un)correlated signal fluctuations in the frequency range above 64 Hz. The finding that UHD recordings yield significantly higher ndRMS than HD recordings is attributed to the amount of tissue sampled by each electrode. These results suggest that ECoG densities with submillimeter electrode distances are likely justified.

**Supplementary Information:**

The online version contains supplementary material available at 10.1007/s12021-024-09689-z.

## Introduction

The advent of noninvasive neuroimaging techniques over recent decades resulted in considerable efforts to elucidate the topographical organization of human sensorimotor brain functions (Huang & Sereno, 2018). Functional magnetic resonance imaging (fMRI) in particular has led to a better understanding of the topographical organization of the sensorimotor cortex (Gordon et al., 2023; Kwong et al., 1992; Meier et al., 2008; Schellekens et al., 2018), but there is no consensus on the exact somatotopic order of and the overlap between body part representations. Increasing the imaging resolution may provide new insights, but fMRI remains limited in temporal resolution and by vascular confounds (Murphy et al., 2013). Therefore, complementary techniques are required to better understand the topographical organization of the sensorimotor cortex.

Electrocorticography (ECoG) may provide the required level of temporal and spatial resolution. For ECoG, typically subdural electrodes are used that are arranged in grids or strips and are generally placed for diagnostic purposes in epilepsy patients (Branco et al., 2023; Lesser et al., 2010). Standard clinical ECoG grids have a center-to-center inter-electrode distance (IED) of 10 mm and an exposed diameter of 2.3 mm, whereas high-density (HD) ECoG grids, which are increasingly used for research, have an IED of 3 to 5 mm and an exposed diameter of about 1 mm. Previous studies have indicated that individual HD ECoG electrodes record significantly distinct sub-centimeter activation patterns (Flinker et al., 2011), and suggest that ultra-high-density (UHD) ECoG may capture even more spatial detail (Chang, 2015; Freeman et al., 2000; Slutzky et al., 2010). Given the newly emerged methods of manufacturing ECoG grids, resolutions below 1 mm are now also feasible (Chiang et al., 2021; Ganji et al., 2018; Khodagholy et al., 2015). By further decreasing the IED to values below 1 mm and the electrode diameter to values below 0.25 mm, the epicortical signals could theoretically start to approach the physiological properties of the local field potential (Bockhorst et al., 2023) and action potentials (Bockhorst et al., 2018; Khodagholy et al., 2016). Conceivably, UHD ECoG may therefore help to unveil the topographical organization of the human sensorimotor cortex.

However, decreasing ECoG IED may lead to spatial oversampling, wherein individual electrodes do not contribute unique information. Typically, spatial oversampling is estimated in terms of the overlap between ECoG electrode signals, measured as the correlation coefficient between pairs of electrodes at specific IEDs (Duraivel et al., 2023; Kellis et al., 2016; Khodagholy et al., 2016; McCarty et al., 2022; Menon et al., 1996; Muller et al., 2016; Rogers et al., 2019). While the correlation between electrode signals is affected by the physical distance between electrodes and by the frequency content of the signals, this metric does not provide a quantitative estimation of the amount of information that is not shared between electrode signals. The degree of shared information in adjacent electrodes is determined by two phenomena, being 1) the physical properties of brain tissue that allows propagation of electrical potentials from a source to surrounding tissue (volume conduction), and 2) the electrophysiological properties of populations of neurons (Dubey & Ray, 2016). While electrophysiological information is of interest for ECoG research on brain function, volume conduction is considered a confounding factor (Nunez & Srinivasan, 2006). Assessing the amount of information that is not shared between electrodes would require the removal of any shared information related to volume conduction.

In the current study we aim to quantify the relationship between ECoG density and amount of non-shared neurophysiological information between electrode pairs, as a means of estimating the most efficient distance between electrodes. We simultaneously recorded HD and UHD ECoG in six participants who underwent resection surgery. We confined the majority of our analysis to resting-state data (Ko et al., 2013; van den Heuvel & Hulshoff Pol, 2010a, b) to avoid the confound of task-induced activity in terms of co-activated regions of unknown location and size. To quantify non-shared neurophysiological activity between pairs of electrodes, we developed a new metric, the normalized differential root mean square (ndRMS). We computed the ndRMS between pairs of ECoG electrodes as a function of IED. Since ECoG exhibits distinctive features across frequencies, notably narrow-band low-frequency oscillations and broad-band high-frequencies (Crone et al., 1998a, b), representing different generating sources (Miller et al., 2009b), we quantified non-shared information across frequency bands.

## Material & Methods

### Participants

We simultaneously recorded HD and UHD ECoG data of six participants undergoing resection surgery, who were either awake (*n* = 4) or under general anesthesia (*n* = 2, see Table 1 for details). All participants gave written informed consent to participate in the current study, which was approved by the medical ethical committee of the University Medical Center Utrecht, in accordance with the Declaration of Helsinki for medical research involving human participants (2013). HD and UHD grids were placed on sensorimotor cortex, in some cases using pre-surgical functional MRI (Hermes et al., 2012).

**Table 1:** Participant characteristics and implantation information

*Participant ID*	*S1*	*S2*	*S3*	*S4*	*S5*	*S6*
*Age*	54	66	63	40	43	44
*Gender*	Male	Female	Male	Male	Female	Male
*State during data collection*	Awake	Awake	Awake	Awake	General anesthesia	General anesthesia
*Included electrodes HD grid (per recording)*	1) 33/96*	1) 38/96 2) 54/96 3) 84/96	1) 67/96	1) 81/96 2) 81/96	N.A	125/128
*Included electrodes UHD grid (per recording)*	1) 19/32	1) 28/32 2) 27/32 3) 29/32	1) 26/32	1) 22/32 2) 28/32	1) 28/32 2) 25/32 3) 25/32	1) 28/32
*Implanted hemisphere*	Left	Right	Left	Left	Right	Right
*HD grid placement*	1) Inferior postcentral gyrus, superior temporal gyrus	1), 2) Inferior pre and postcentral gyrus 3) Superior pre and postcentral gyrus	1) Inferior pre and postcentral gyrus	1), 2) Inferior pre and postcentral gyrus	N.A	1) Inferior pre and postcentral gyrus
*UHD grid placement*	1) Inferior postcentral gyrus	1), 2), 3) Inferior precentral gyrus	1) Inferior precentral gyrus	1), 2) Inferior postcentral gyrus	1) Prefrontal cortex 2) Temporal fissure 3) Inferior precentral gyrus	1) Inferior precentral gyrus
*Resection type*	Tumor	Tumor	Cavernoma	Epilepsy	Epilepsy	Epilepsy

The participants who were awake during data collection performed one or more repetitions of speech and/or hand motor tasks. Included electrodes are shown per applicable recording method and repetition of recording

*N.A.* not applicable

*Data was recorded with a 128-channel grid, but only 96 channels were connected to the amplifier

### Electrode Grids

**Fig. 1:**
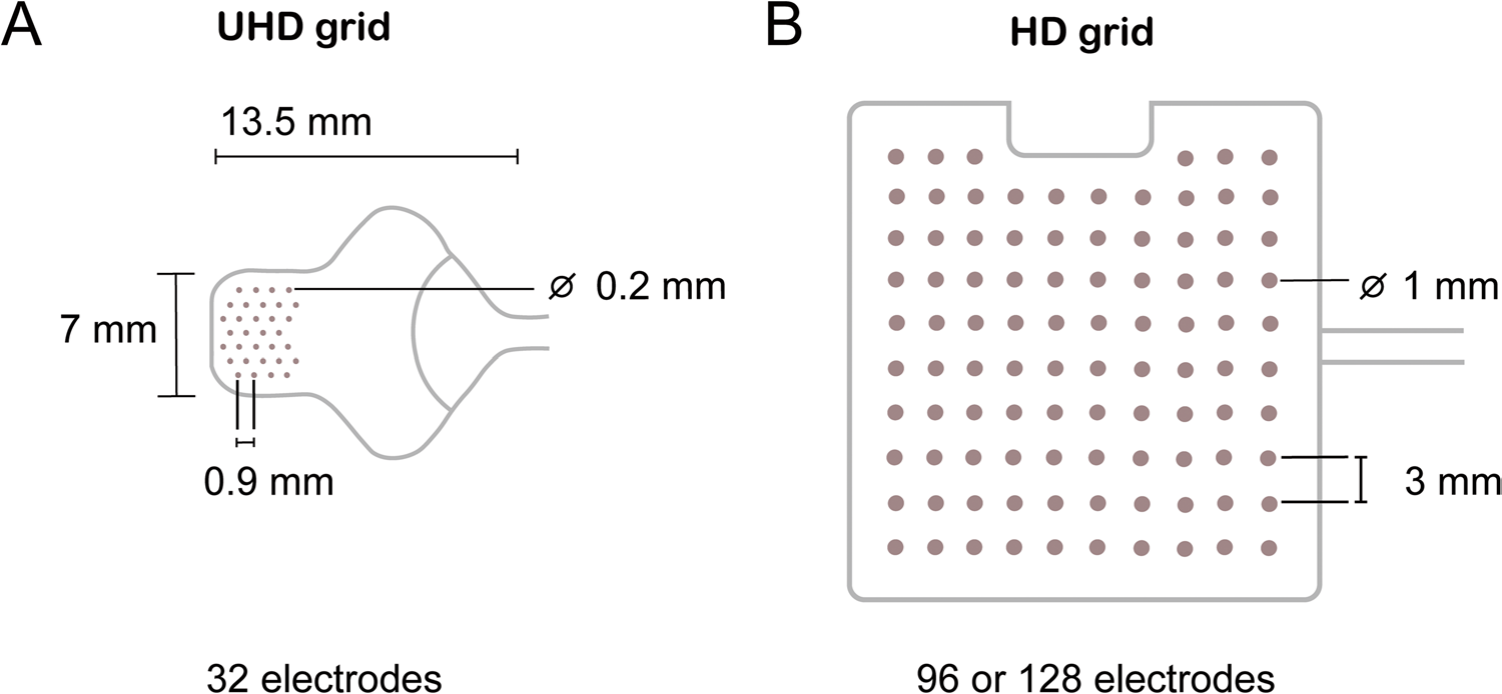
Sketches and measures in scale of (**A**) the UHD grids (CorTec Neuro, Freiburg, Germany) and (**B**) the 96-channel HD grid (Ad-Tech Medical, Oak Creek, USA). The 128-channel HD grid (PMT Corporation, Chanhassen, USA) had an 8 by 16 layout with the same IED and exposed diameter

The UHD grids (CorTec Neuro, Freiburg, Germany) consisted of 32 platinum-iridium electrode contacts at 0.9 mm IED and 0.2 mm exposed diameter, embedded in a silicone sheet (Fig. 1A). The HD grids consisted of 96 platinum electrode contacts (Ad-Tech Medical, Oak Creek**,** USA) or 128 platinum-iridium electrode contacts (PMT Corporation, Chanhassen, USA) at 3 mm IED and 1 mm exposed diameter, embedded in a silicone sheet (Fig. 1B).

### Intraoperative Recordings

The size and location of the craniotomy was determined solely by the clinical plan. The recordings took place at a convenient time during the surgery, before or after resection. A ground electrode was placed on the participant’s forehead, and a reference electrode on the mastoid. The neurosurgeon positioned the electrode grids on exposed or accessible healthy brain tissue for 5 to 15 min, and a sterilized electrode cable connected the sterile electrode grids to a signal amplifier (Blackrock Neurotech, Salt Lake City, USA). The amplifier recorded the two grids in parallel with a sampling frequency of 2000 Hz and a digital bandpass filter of 0.3 to 500 Hz.

### Resting-State Data

During awake surgeries, the participants (*n* = 4) performed one or more repetitions of an overt speech task, such as articulating different phonemes, or a hand movement task. We extracted rest and active trials from the task data, where a rest trial was defined as the period of half the inter-stimulus interval up to stimulus presentation, and an active trial was the period from stimulus presentation up to half the inter-stimulus interval. Depending on the task, these trials lasted between 1 and 1.75 s. For the main objective of this study, only the rest trials were used to quantify the non-shared information between electrodes. To illustrate differences in activity between (adjacent) electrodes in one participant, we used both rest and active trials to compute the response to the task per electrode. For participants who were under general anesthesia (*n* = 2), we analyzed the entire recording period as a single stream.

### Signal Pre-Processing and Power Extraction

Signal processing was conducted with MATLAB (MathWorks, Natick, USA) using the FieldTrip toolbox (Oostenveld et al., 2011). If intraoperative pictures showed HD channels laying on top of the UHD grid, these HD channels were excluded from the analysis. Channels were considered bad if they showed a flat signal, abnormal amplitude, excessive line noise and/or outlier data points (adapted from (Liu et al., 2015)), and were excluded from further analysis. The selection of bad channels was confirmed by visual inspection of the raw and power spectral signals. We removed line noise (50 Hz and harmonics) and a 24 Hz peak (and harmonics) encountered in the operating room, from the remaining channels. The 50 Hz and 24 Hz components were removed with a 4th order Butterworth band stop filter at the cut-off frequencies $\pm$ 1 Hz. We re-referenced the data using a common median reference filter to remove noise and artefacts and other activity shared by all electrodes (Liu et al., 2015). We extracted frequency information from eight frequency bands, using standard wavelet decomposition levels (1–4 Hz, 4–8 Hz, 8–16 Hz, 16–32 Hz, 32–64 Hz, 64–128 Hz, 128–256 Hz and 256–499 Hz). The bandwidths correspond to a geometric sequence (factor 2) and approximate the frequency bands that are deemed relevant in electrophysiology (delta, theta, alpha, beta, lower range of the high frequency band and higher ranges, including the typical ECoG high-frequency band). For the ndRMS, we used a 3rd or 4th order Butterworth bandpass filter (for 1–4 Hz / 4–8 Hz and higher frequency bands, respectively) and also computed ndRMS for unfiltered data. For the correlation analysis, we used a Morlet wavelet for time–frequency decomposition.

### Normalized Differential Root Mean Square (ndRMS)

The root mean square (RMS) is a commonly used metric to calculate the overall magnitude of an alternating (neuro)physiological signal. Signal amplitude changes with frequency band, because of the power law scaling of signal amplitude over frequency (Miller et al., 2009a), and with electrode size, because of the higher impedance of smaller electrodes (Fan et al., 2021; Ganji et al., 2017; Tolstosheeva et al., 2015). These amplitude changes can hamper a fair comparison between frequency bands and properties of electrodes. With the ndRMS, we calculate the RMS of the difference between *normalized* bandpass-filtered signals, thereby effectively eliminating the influence of electrode size and frequency band on the signal amplitudes. For this, we first normalized all samples in the bandpass-filtered time domain of the pre-processed rest trial ($${trial}_{n})$$ by the mean ($${\mu }_{trial}$$) and standard deviation ($${\sigma }_{trial}$$) over all samples of that trial, yielding normalized trial signals ($$tria{l}_{norm}$$) with a mean value of 0 and a standard deviation of 1:

$$tria{l}_{norm}\left(n\right)= \frac{{trial}_{n}- {\mu }_{trial}}{{\sigma }_{trial}} ,$$

where *n* corresponds to each time sample in the trial. Any remaining differences between trials lay in the phase or shape of the signal. Next, we created differential signals by subtracting the normalized signals within all electrode pairs in the grid ($\Delta {trial}_{norm}(n)$). Then, we calculated the RMS of the normalized differential signals per trial ($tria{l}_{RMS}$), yielding one value per trial:

$$tria{l}_{RMS}= \sqrt{\frac{1}{N}\sum_{n=1}^{N}{\Delta tria{l}_{norm}(n)}^{2}},$$

where *N* is the total number of samples in the normalized trial.

If the neural signals would exhibit only sinusoidal properties, the theoretical minimum ndRMS would be 0, i.e., completely identical signals. When the signals contain oscillations that are out of phase, the ndRMS is inflated by the difference in amplitude at a specific frequency, which holds for all oscillatory frequencies in the signal. The ndRMS for two sinusoidal oscillations at phase difference ± π would then be 2, which is thus the maximum value the ndRMS can reach (Sup. Fig. 1A). In contrast, when the neural signals would consist solely of white noise, the ndRMS would be $\sqrt{2}$ (Sup. Fig. 1B). Thus, we assume neural signals resulting in ndRMS values between $\sqrt{2}$ and 2 to contain oscillatory activity.

For awake participants, we calculated the RMS for each trial and then took the median over trials, as the median is less sensitive to outlier trial values than the mean. For participants under general anesthesia, the RMS was calculated once, across the entire recording. We then calculated the mean ndRMS over all equidistant electrode pairs, for all possible IED’s, for each participant. Only IED’s that included ten or more pairs were included for further analysis. We subsequently averaged the ndRMS over task repetitions for each participant, for each IED and for all eight frequency bands and unfiltered data. In addition, we analyzed the ndRMS for each IED and frequency band averaged across all participants, but only for IEDs present in two or more participants.

### Comparing the ndRMS Between HD and UHD ECoG Grids

To compare the effect of IED on the ndRMS between HD and UHD grids, we calculated the median ndRMS over all frequency bands to obtain results per IED. Since HD and UHD grids differ in standard IED, only two IEDs are comparable between the grids: 3.0 mm in the HD grid versus 3.1 mm in the UHD grid, and 4.2 mm in the HD grid versus 4.1 mm in the UHD grid. At these IEDs, we compared the median ndRMS over all frequency bands with Bonferroni-corrected two-sided Wilcoxon rank-sum tests (at *p* < 0.05), since ndRMS values over frequency bands did not follow the normal distribution. Next, to illustrate that the difference between HD and UHD grids at 3 and 4 mm IED was consistent over frequency bands, we present the ndRMS per frequency band at these IEDs, both averaged over participants and per individual participant.

### Distance-Averaged Correlation

To compare our results with the findings of previous human UHD ECoG studies (Duraivel et al., 2023; Kellis et al., 2016; Khodagholy et al., 2016; Rogers et al., 2019), we computed pairwise correlations across electrodes. Pearson correlation coefficients between all pairs of electrodes on a grid were extracted for each rest trial and frequency band. We then averaged the correlation coefficients over all rest trials, followed by averaging for each IED, provided that there were at least ten pairs. We subsequently averaged the ndRMS over task repetitions for each participant, for each IED, and for all eight frequency bands and unfiltered data. In addition, we analyzed the correlation coefficient for each IED and frequency band averaged across all participants (for IEDs present in two or more participants).

### Neural Response to an Overt Speech Task

To illustrate the spatial spread of neural activity underneath the HD and UHD electrodes, we analyzed data recorded during a task where S4 pronounced a set of 99 words. We focused on the 64–128 Hz band, since that is generally regarded as a measure of local neural activity (Miller et al., 2009b; Siero et al., 2014). A direct statistical comparison between grids was not deemed meaningful given the grids recorded from different cortical patches. A rest trial was defined as 1.5 s before stimulus presentation up to stimulus presentation. An active trial was defined as the period between stimulus presentation and 1.5 s after stimulus presentation. We correlated the mean power in the 64–128 Hz band per trial to a binary task regressor (0 for rest, 1 for active) using Pearson’s correlation coefficient. The coefficient of determination was calculated by squaring the correlation coefficient. P-values were computed using a t-distribution. The threshold for significance was Bonferroni corrected for the number of channels in the analysis of each grid. We determined the exact location of the HD grid of S4 using the Gridloc method (Branco et al., 2018), and visually determined the location of the UHD grid using intraoperative pictures taken of the grids *in situ*.

## Results

**Fig. 2:**
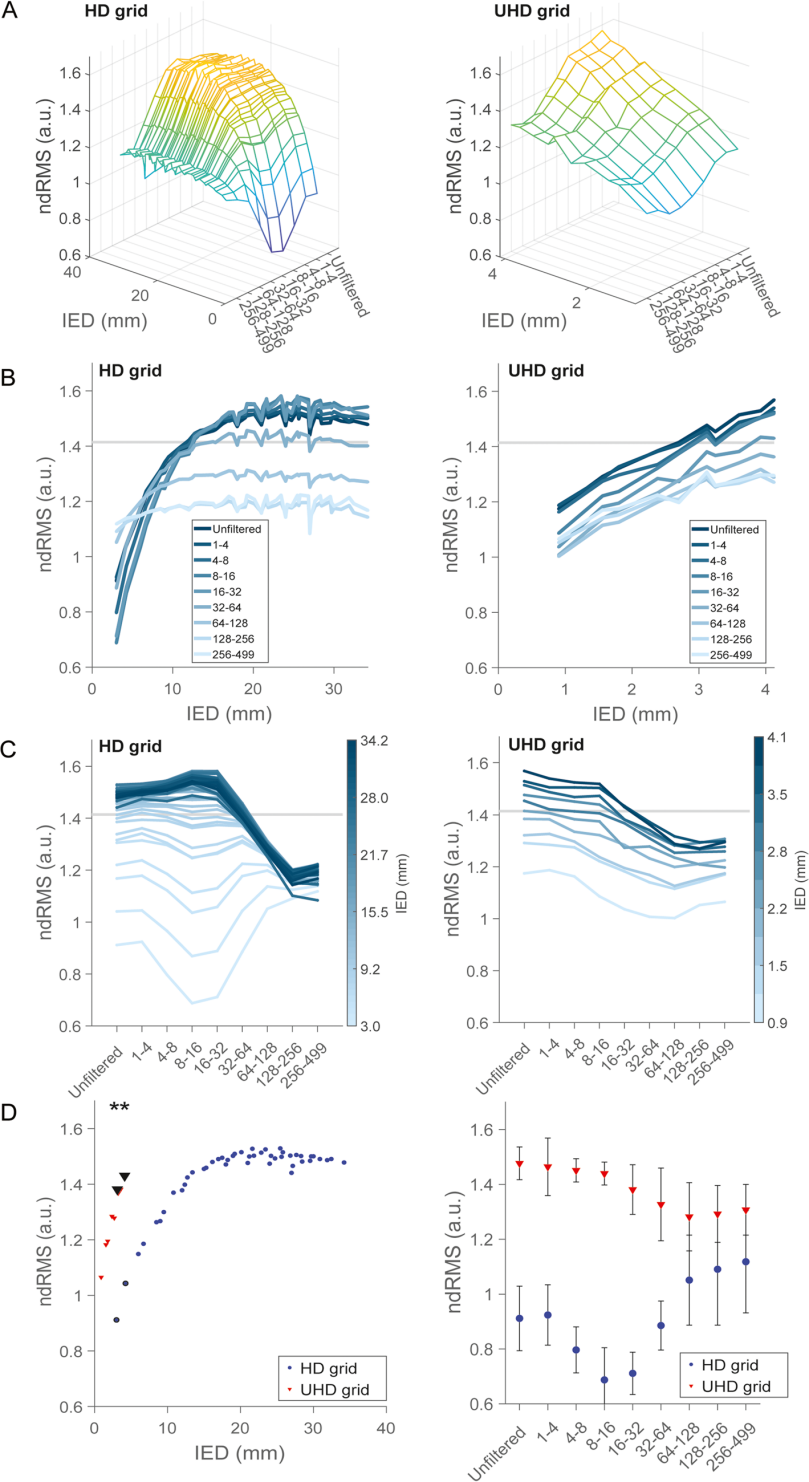
Average ndRMS over all participants. **A** ndRMS represented over IED and frequency bands (in Hz) for the HD (left column) and UHD grid (right column). The color scale indicates ndRMS magnitude. **B** ndRMS as a function of IED and (**C**) frequency bands (in Hz). Horizontal lines in B and C denote $\surd 2$. **D** Direct comparison between HD (red) and UHD (blue) grids. The left column shows the median ndRMS per IED over all frequency bands, where asterisks denote significant difference between medians (Wilcoxon rank-sum test, *p* < 0.0001, W = 45, at both IEDs). The right column shows the average ndRMS over all participants (± standard deviation), per frequency band (in Hz), at 3 mm IED

The ndRMS for the unfiltered signal decreased with decreasing IED (Fig. 2A), which was consistent across grids and participants (Sup. Fig. 2). For the HD grids, as IED declined the ndRMS remained stable until 15 mm IED, where it started to decrease (Fig. 2B, left panel). For the UHD grids, the ndRMS decreased steadily across all IED’s (Fig. 2B, right panel), which indicates that at distances of 4 mm and below, electrodes increasingly shared information. This was in line with the HD grid data (Fig. 2A, left panel).

The effect of IED on ndRMS was most prominent for lower frequencies but was present up to 128 Hz for HD and in all frequency bands for UHD grids. Notably, frequency bands in the classic oscillation range up to the beta band (16–32 Hz) exhibited ndRMS on average above $\sqrt{2}$ (Fig. 2C), in line with our prediction for oscillations (Sup. Fig. 1A), when IEDs were above approximately 15 mm for HD grids and 4 mm for UHD grids (Fig. 2B). For frequencies above 64 Hz, the ndRMS remained below $\sqrt{2}$, the maximum value for white noise uncorrelated between electrode pairs (Sup. Fig. 1B). Of note, ndRMS values remained above 1 for all IED’s and frequency bands for the UHD grids, whereas ndRMS values for HD grids dropped relatively fast with declining IED for the frequency bands that contain oscillations (Fig. 2B). The ndRMS profiles were consistent across participants (Sup. Fig. 2).

Since electrodes in HD and UHD grids were spaced equally far apart at two IEDs, i.e. 3 and 4 mm, a direct comparison between grids was possible at these points. For these IEDs, the UHD grid recorded significantly larger ndRMS (median across frequency bands) than the HD grid (Wilcoxon rank-sum test, *p* < 0.0001, W = 45, at both IEDs; Fig. 2D, left column). For both IEDs, this difference is consistent over frequency bands (Fig. 2D, the right column) and participants (see also Sup. Figs. 3 and 4).

**Fig. 3:**
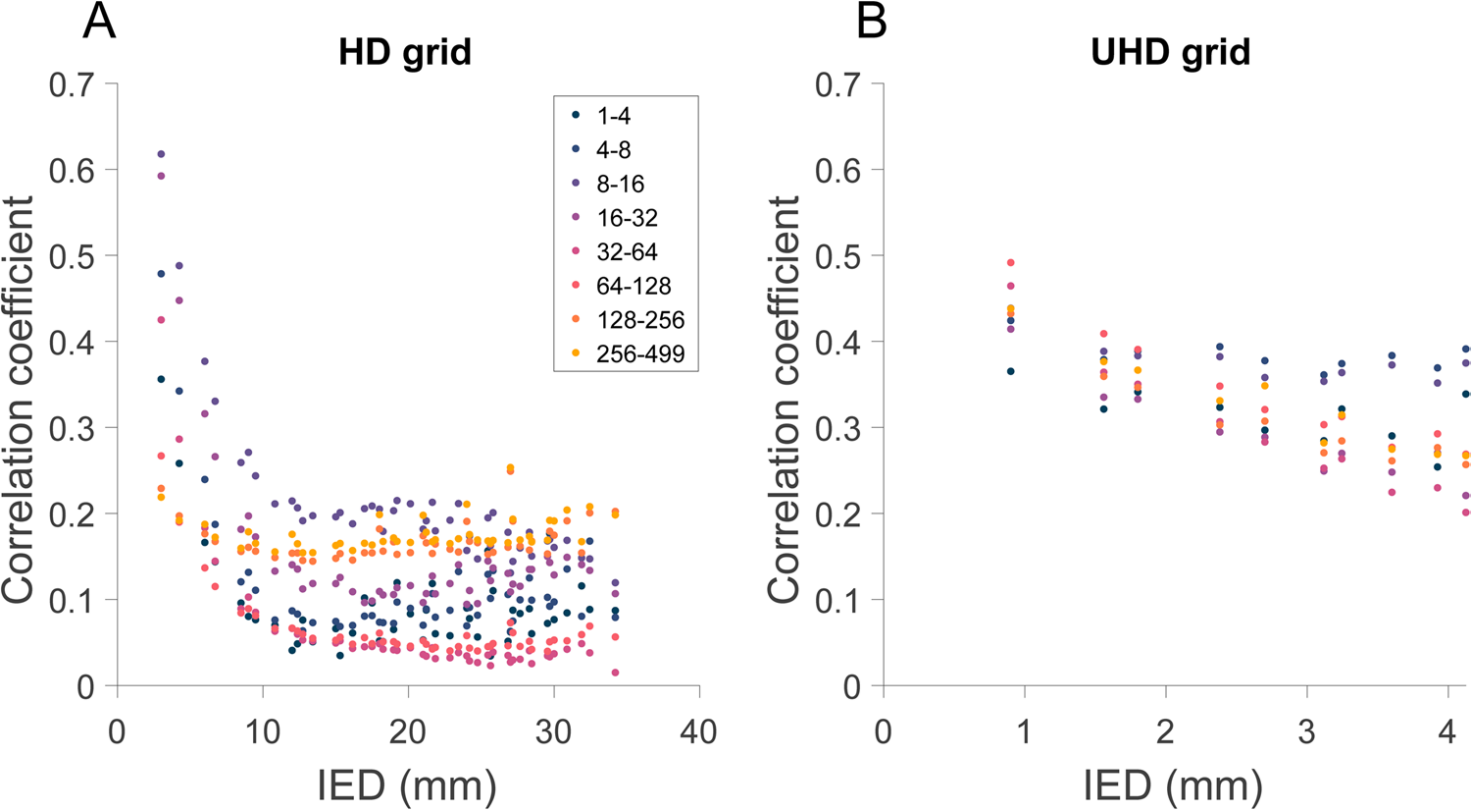
Correlation as a function of IED, averaged over all participants for (**A**) HD grid and (**B**) UHD grid. Color coding indicates frequency band (in Hz)

The inter-electrode correlation decreased with increasing IED in both HD and UHD grids in all frequency bands (Fig. 3). For HD grids, the decline in correlation with increasing IED appears to differ for frequency bands below versus above 128 Hz, which was not apparent in UHD grids.

**Fig. 4:**
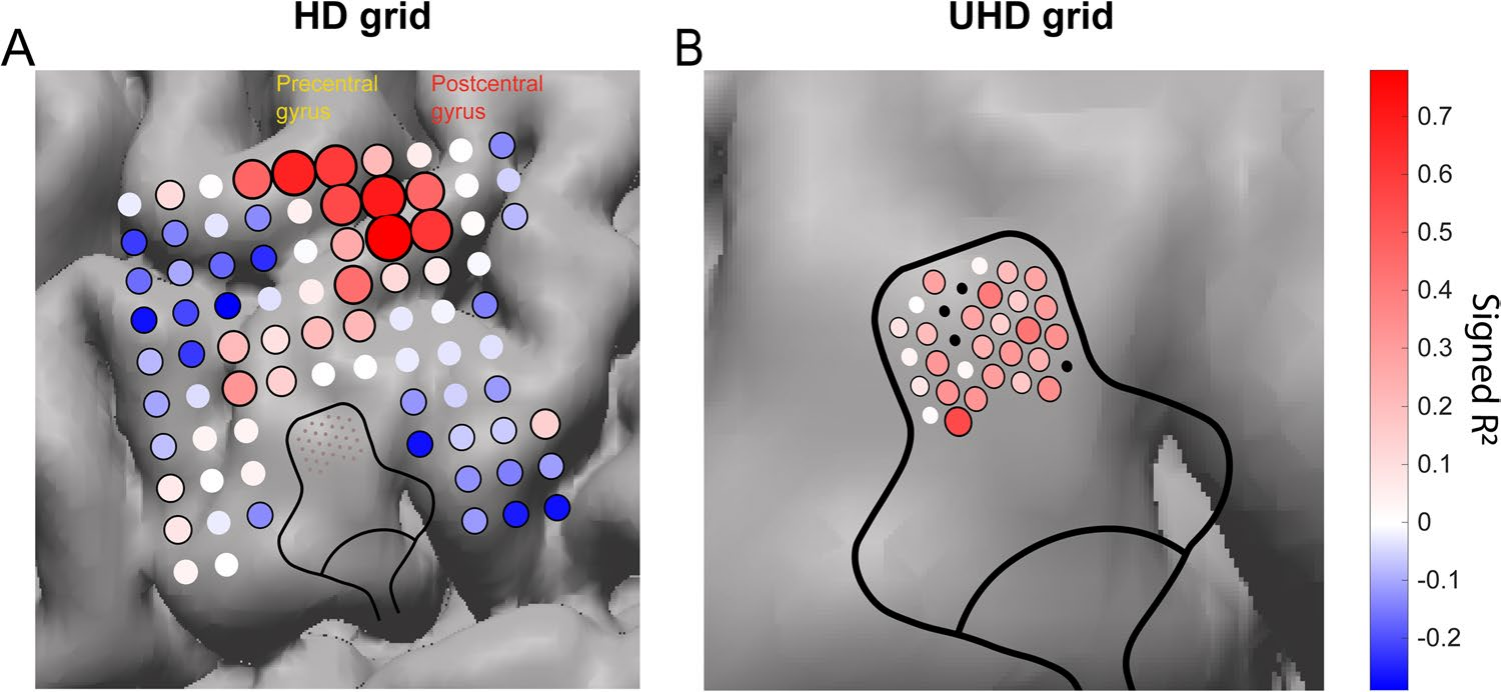
High-frequency band (64–128 Hz) response regressed to an overt speech task for S4. The coefficient of determination is plotted on the brain of S4. Electrodes excluded from analyses due to poor signal are indicated in black. Electrode color corresponds to the correlation value as shown in the colormap. The electrode radius increases with the magnitude of the coefficient. Electrodes that respond significantly to the task are circumvented in black. **A** Signed r-squared values of the HD grid. Electrodes that lay on top of the UHD grid are not shown. **B** Signed r-squared values of the UHD grid

The high-frequency band (64–128 Hz) response in the HD grid of S4 was evident in both pre- and postcentral gyrus (Fig. 4A), exhibiting a coherent pattern of positive and negative R^2^ values. The response in UHD electrodes, which was positioned on the post-central face area, also varied across electrodes, but here we observed no negative responses. Yet, a clear variation was seen, with some electrodes that showed no response (R^2^ of zero) lying adjacent to electrodes with a strong response (Fig. 4B).

## Discussion

In this study we assessed the relationship between IED and the amount of non-shared neurophysiological information in the sensorimotor cortex. We simultaneously recorded HD and UHD ECoG during surgery and introduced the ndRMS as a measure of non-shared information between electrode pairs. We found that 1) the ndRMS decreased linearly with decreasing IED from 15 mm IED to the minimum separation distance of the UHD grids, 0.9 mm, 2) at sufficiently large IED, the ndRMS of frequencies below 32 Hz exceeded the value $\sqrt{2}$, and 3) the ndRMS was higher in UHD grids than HD grids for electrodes spaced equally far apart. Overall, the ndRMS revealed that UHD ECoG recorded unique neurophysiological information up to the minimum IED, in all frequency bands.

Our finding that continued linear decrease of ndRMS with decreasing IED in all frequency bands did not level out, nor approached zero, suggests that a considerable difference between neural signals can be measured at a submillimeter resolution. This agrees with the variable response to the task between adjacent electrodes in the high-frequency band, as recorded with the UHD grid of S4, and is in line with the previously proposed practical target range of 0.5 – 1 mm IED for new ECoG grids (Chang, 2015). Such recordings contribute to the mapping of increasingly detailed neural dynamics. Paulk et al., (2021) could for example distinguish three types of waveforms at microscale level with UHD ECoG. Furthermore, smaller electrodes are predicted to record from more shallow layers than larger electrodes (Wodlinger et al., 2011), thus UHD ECoG may capture spiking activity in layer I (Bockhorst et al., 2023; Khodagholy et al., 2015; Suzuki & Larkum, 2017).

At sufficiently large IED the ndRMS of frequencies below 32 Hz exceeded the value $\surd 2$ on average. For those frequencies, the ndRMS may be dominated by phase differences in oscillatory activity between electrode pairs. For frequencies above 32 Hz, broadband activity dominates the power spectrum, which is thought to primarily consist of asynchronous activity (Miller et al., 2009b). In our study, a decrease in the IED coincided with a decrease in the ndRMS to values smaller than $\surd 2$. This could indicate a decline of oscillation-phase difference between electrodes for low frequencies, and increasingly correlated asynchronous signals for the high-frequency band.

Our finding that the ndRMS was higher in UHD grids than HD grids when the electrodes are spaced equally far apart likely reflects an effect of electrode size, since acquisition was performed with the same amplifier and reference electrode. The difference in ndRMS supports the notion that smaller electrodes can record phenomena that larger electrodes cannot. Indeed, heterogenous uncorrelated activity may be attenuated in large electrode grids, as they record the average activity over large populations of neurons (Lindén et al., 2011), and UHD ECoG records higher power in frequencies above 40 Hz than clinical grids (Duraivel et al., 2023; Khodagholy et al., 2016).

### ndRMS: a Method to Quantify the Non-Shared Information Between Electrodes

In the current study, correlation coefficients were lower for the UHD grids than in previous studies (Duraivel et al., 2023; Khodagholy et al., 2016; Rogers et al., 2019). These lower values may have been due to the common average referencing we applied (in our case median rather than average): a previous study found that common average referencing considerably decreased the range of correlation coefficients within UHD ECoG (Rogers et al., 2019). Signal common to all electrodes can be injected from external sources, such as line noise, or from the reference electrode in the montage. Neurophysiological sources may also affect all electrodes (either by volume conduction or axonal projections), but since the phase of generated oscillations or non-oscillatory perturbations may vary with distance from a source or length of axonal projections, these effects are not likely removed by common average re-referencing and thus contribute to the ndRMS.

### Limitations & Future Directions

Recording in the operating room provided the unique opportunity to simultaneously record HD and UHD ECoG in six human participants. Yet, there were several limitations worth mentioning. First, the recordings were made during resection surgeries, with little time to optimize the placement of the grids and the signal quality of the recordings. The recorded signals may have also been contaminated by equipment in the operating room (other than the 24 Hz signal that we filtered out). A second limitation relates to the fact that HD grids tend to cross over sulci. Electrodes on either side may be spatially close, but cortically far apart. Indeed, correlations between electrode pairs located on the same gyrus are reportedly higher than between electrodes across sulci (Menon et al., 1996; Muller et al., 2016). Since our accuracy of determining grid and electrode positions relative to sulci was limited to photographs on which sulci were difficult to delineate, we did not distinguish between such electrode pairs. This may have artificially increased the ndRMS values averaged across all equidistant electrode pairs. However, visual inspection of data from S4, the only participant where sulci were clearly visible under the HD grid, at IED’s of 3 and 4 mm, did not provide any indication of distinct separate distributions of ndRMS values that would support this notion. UHD grids did not lay over sulci, and thus were not affected. Future studies may replicate our study in a different recording environment that allows for such distinction (e.g., with a presurgical MRI scan and postsurgical computed tomography scan), such as the epilepsy monitoring unit, and investigate how the ndRMS is affected by sulci and how it differs between anatomical regions. Replication of our study in less restrictive recording environments will enable larger sample sizes to assess generalizability of our findings including task-induced activation, explore individual differences in cortical organization, and further assess the potential benefits of UHD ECoG in research and clinical settings.

The ndRMS may be employed more broadly, such as in modeling studies, to exactly parse out how the recording of distinct neural signals by ECoG electrodes is influenced by electrode size or inter-electrode distance. Conceptually, ndRMS may be helpful in investigating the contribution of single neural sources to recorded signals. As the ndRMS eliminates any amplitude differences between recordings, it may quantify the unique signal properties of different recording modalities. This is particularly relevant while comparing the intracortical local-field potential as recorded with microelectrode arrays with the epicortical signals as recorded with UHD ECoG. Another exciting application of the ndRMS may be to investigate the speed of neural transmission. This is a fundamental property of the temporal organization of the cortex (Buzsáki et al., 2013), but is difficult to study with intracranial methods. A recent study using cortico-cortical evoked potentials during ECoG recordings found that transmission speed increases with age until 35 years, with short range-connections such as within the sensorimotor cortex reaching maximum speeds of about 2–3 m/s, and long-range connections reaching 3–6 m/s (van Blooijs et al., 2023). Postulating oscillatory activity that originates from an identifiable, distinct neural source, the ndRMS may indicate the phase difference between electrodes, with 0 meaning complete overlap and 2 meaning that the signals are in antiphase. By identifying how the phase differences change as a function of IED, one may make inferences about the transmission speed of cortico-cortical connections underlying the ECoG grid.

### Conclusion

We evaluated how increasing ECoG density affects non-shared neurophysiological information between electrodes pairs. We found that non-shared information between ECoG electrodes increased with IED up to 15 mm after which it stabilized. Frequencies below 32 Hz exhibited prevalent out-of-phase oscillatory activity, and when ECoG electrodes were spaced equally far apart, UHD ECoG recorded a significantly larger differential signal than HD. These results highlight the potential of increasing ECoG density to further our understanding of the sensorimotor cortex topographical organization.

## Information Sharing Statement

By European law (the General Data Protection Regulation (GDPR)), we are not allowed to publicly share patient data. Sharing data would require setting up a data sharing agreement between our institution and the institution requesting the data.

## Supplementary Information

Below is the link to the electronic supplementary material.

Supplementary file1 (DOCX 1378 KB)

## Data Availability

Data can be made available upon reasonable request from the authors, respecting privacy protection. A function to calculate the ndRMS (and associated documentation) is openly available at https://github.com/UMCU-RIBS/ndRMS/.
