# Supplementary material for: Effect of Electrode Distance and Size on Electrocorticographic Recordings in Human Sensorimotor Cortex
Source: Neuroinformatics. 2024 Oct 9;22(4):707–17. doi: 10.1007/s12021-024-09689-z (PMC11579129; doi:10.1007/s12021-024-09689-z)
Supplement: Supplementary file 1 — Supplementary file1 (DOCX 1378 KB) [file 12021_2024_9689_MOESM1_ESM.docx]

# Supplementary figures


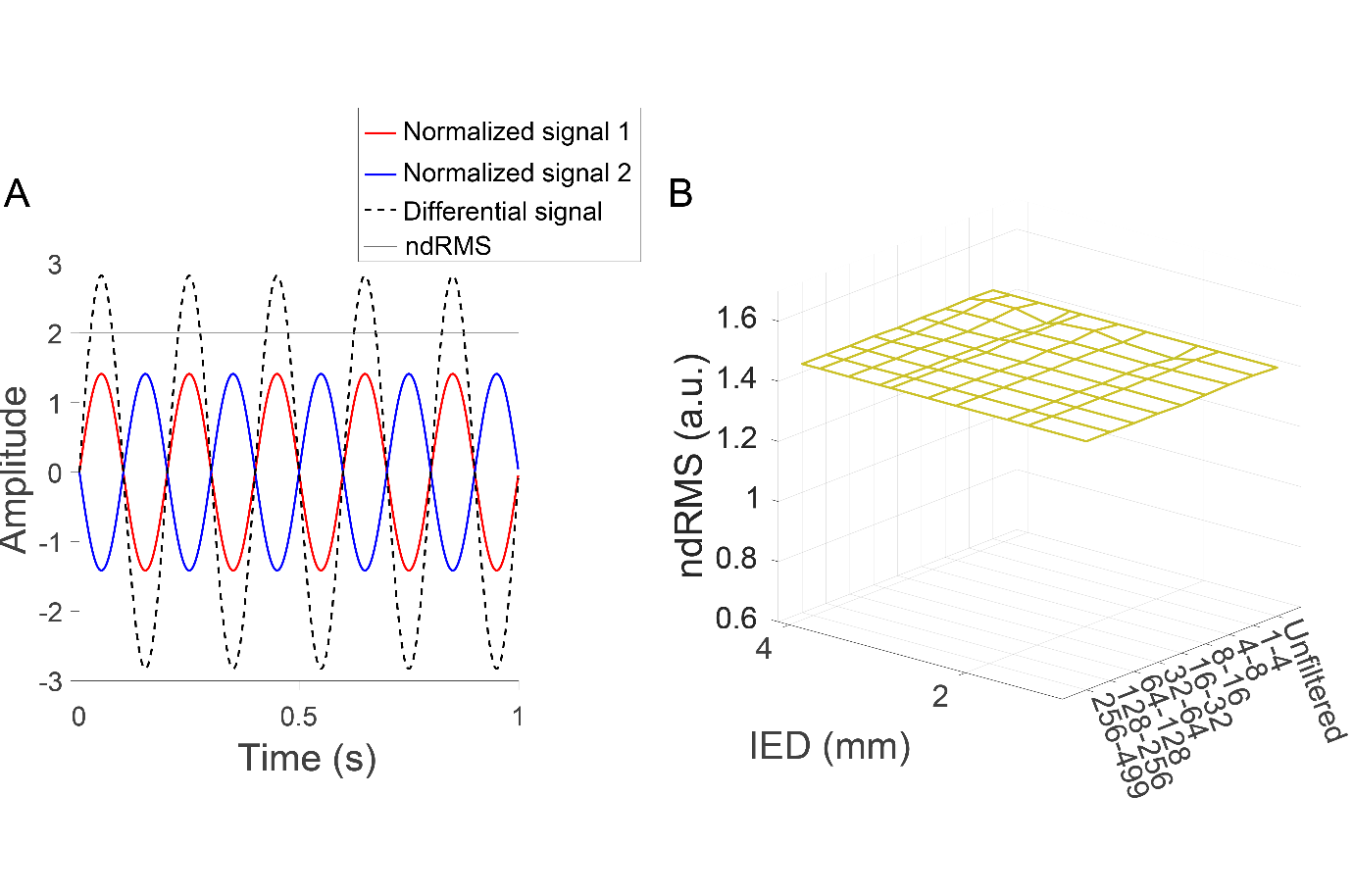


*Sup. Fig. 1 – A) The root mean square of the differential signal of two normalized signals (ndRMS) with oscillatory properties and phase difference ±π. 2 is the maximum value the ndRMS can reach. B) Running white noise through the analysis pipeline of ultra-high-density ECoG results in an ndRMS of* $\surd2$*, across all inter-electrode distances (IEDs) and frequency bands* (in Hz)*.*


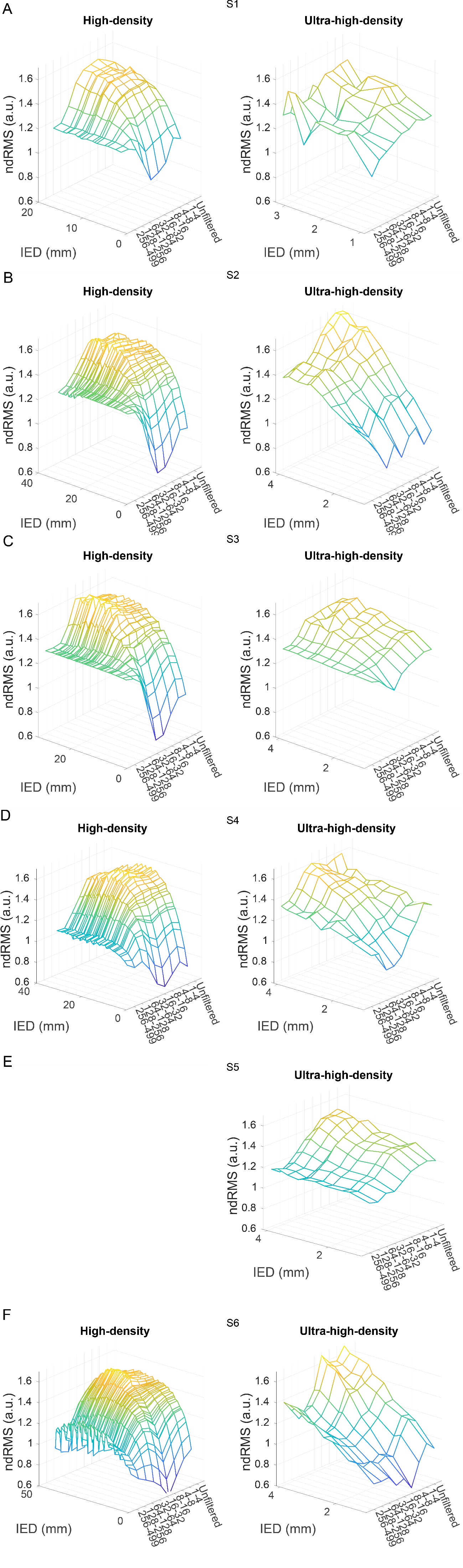


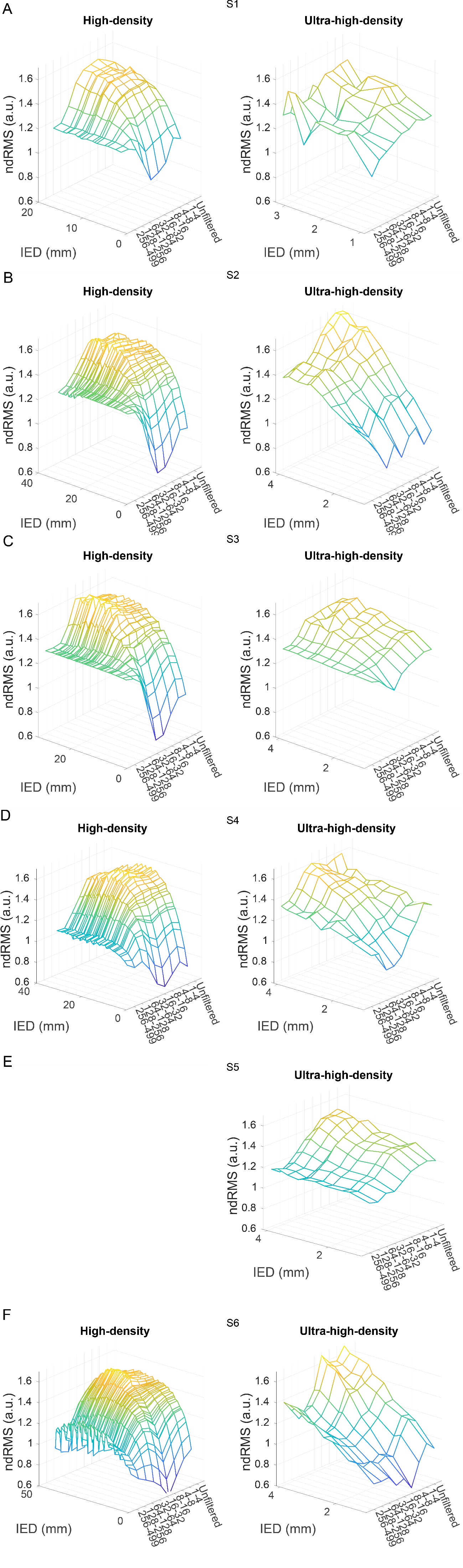


Sup. Fig. 2 – ndRMS for all participants over IED, for different frequency bands (in Hz).


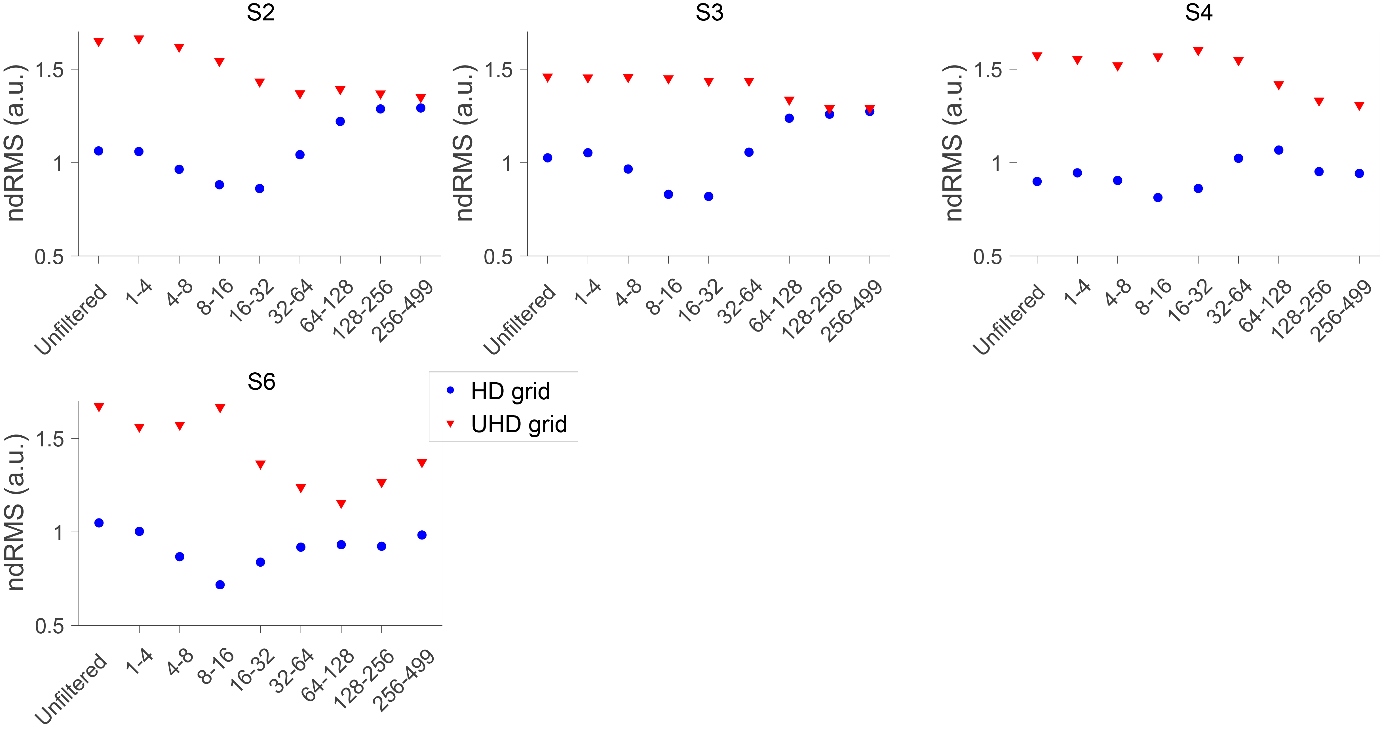

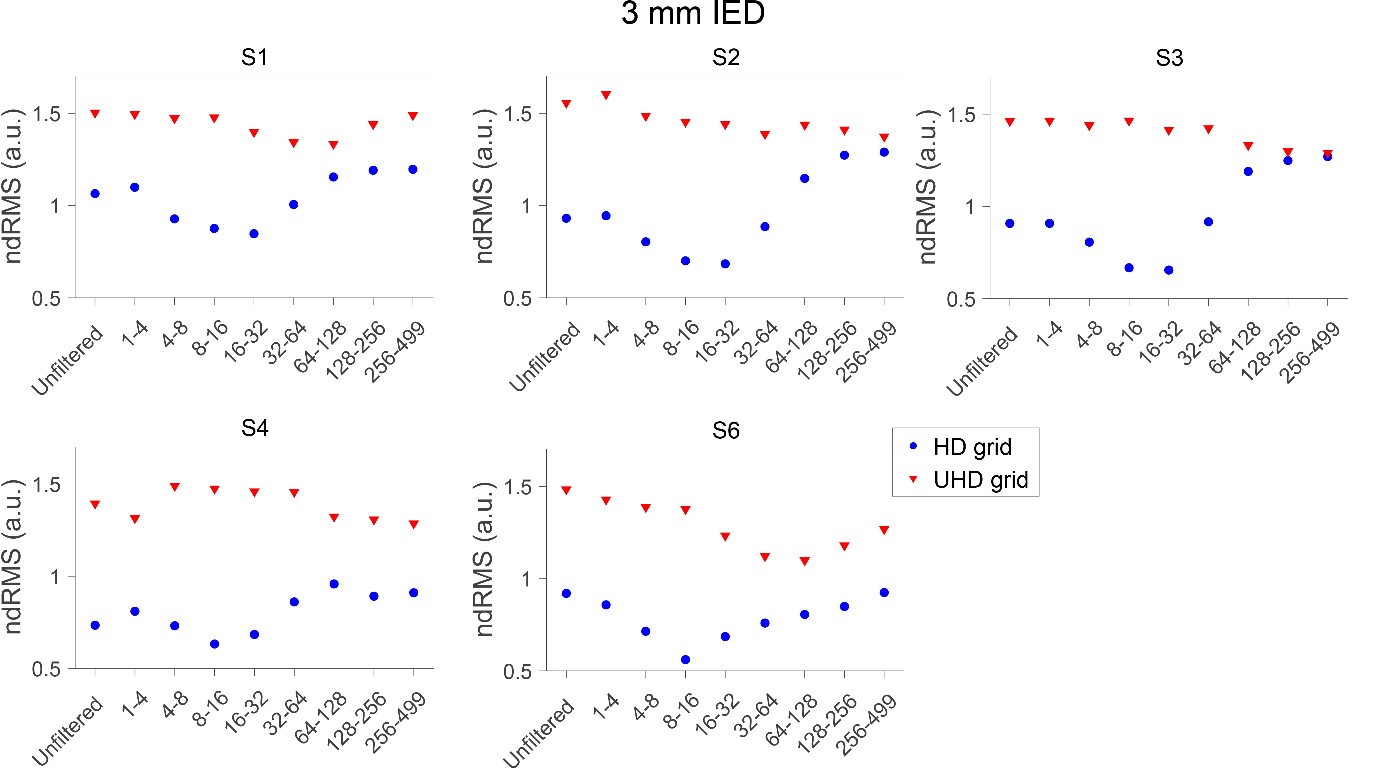


Sup. Fig. 3 – Comparison of the ndRMS at 3 mm IED between HD (blue) and UHD (red) grids for each participant with simultaneous HD and UHD grid recordings.

Sup. Fig. 4 – Comparison of the ndRMS at 4 mm IED between HD (blue) and UHD (red) grids for each participant with simultaneous HD and UHD grid recordings, except for S1, whose UHD grid lacked enough electrode pairs at this IED.
